# Alterations in cellular metabolism under different grades of glioma staging identified based on a multi-omics analysis strategy

**DOI:** 10.3389/fendo.2023.1292944

**Published:** 2023-12-04

**Authors:** Xianlei Yan, Jinwei Li, Yang Zhang, Cong Liang, Pengcheng Liang, Tao Li, Quan Liu, Xuhui Hui

**Affiliations:** ^1^ Department of Neurosurgery, West China Hospital, Sichuan University, Chengdu, Sichuan, China; ^2^ Department of Neurosurgery, Liuzhou Workers Hospital, Liuzhou, Guangxi, China; ^3^ Department of Vascular Surgery, Fuwai Yunnan Cardiovascular Hospital, Affiliated Cardiovascular Hospital of Kunming Medical University, Kunming, Yunnan, China; ^4^ Department of Pharmacy, Liuzhou Workers Hospital, Liuzhou, Guangxi, China; ^5^ Department of Medical Imaging, Liuzhou Workers Hospital, Liuzhou, Guangxi, China

**Keywords:** multi-omics analysis, cellular metabolism, glioma, single-cell, biomarker

## Abstract

Glioma is a type of brain tumor closely related to abnormal cell metabolism. Firstly, multiple combinatorial sequencing studies have revealed this relationship. Genomic studies have identified gene mutations and gene expression disorders related to the development of gliomas, which affect cell metabolic pathways. In addition, transcriptome studies have revealed the genes and regulatory networks that regulate cell metabolism in glioma tissues. Metabonomics studies have shown that the metabolic pathway of glioma cells has changed, indicating their distinct energy and nutritional requirements. This paper focuses on the retrospective analysis of multiple groups combined with sequencing to analyze the changes in various metabolites during metabolism in patients with glioma. Finally, the changes in genes, regulatory networks, and metabolic pathways regulating cell metabolism in patients with glioma under different metabolic conditions were discussed. It is also proposed that multi-group metabolic analysis is expected to better understand the mechanism of abnormal metabolism of gliomas and provide more personalized methods and guidance for early diagnosis, treatment, and prognosis evaluation of gliomas.

## 1 Background

Gliomas are the most common primary intracranial tumors that originate from brain glial cells (1). The grading system and classification criteria for brain gliomas are based on their histological features, cellular heterogeneity, and biological behavior. The 2021 WHO classification has taken a significant step in advancing the role of molecular diagnostics by incorporating critical diagnostic genes, molecules, pathways, and/or combinations into tumor classification (2). In addition, the new grading system emphasizes biological similarities within tumor types. Moreover, by incorporating molecular features alongside histological grading, tumors can be classified at a higher grade when they exhibit specific molecular alterations, even if they show lower-grade histological characteristics. Therefore, molecular characterization may be one of the most important criteria for determining the glioma grade.

The involvement of multi-omics technologies has also facilitated the development of new classifications for gliomas. In recent years, the analysis of DNA methylation profiles has emerged as a powerful classification of central nervous system (CNS) tumors (3–6). Copy number profiles can also be inferred using methylation, such as identifying genetic alterations, including 1p/19q codeletion, the +7/−10 signature, amplifications, homozygous deletions, and patterns indicative of fusion events (7). In addition, DNA methylation analysis serves as an essential reference when targeted therapy and clinical trials require confirmation of specific mutations in patients before treatment.

The prognosis of glioma patients is closely correlated with the grade of gliomas. Grade 1 and 2 gliomas are classified as low-grade gliomas, while grades 3 and 4 are classified as high-grade gliomas (2). Glioblastoma (GBM), the most aggressive type of glioma, is categorized as a grade 4 tumor. However, many low-grade glioma patients can achieve long-term survival with the appropriate treatment. These tumors grow slowly and have relatively minimal invasion into surrounding tissues, leading to a favorable prognosis. Some patients may even experience survival for several decades (2). High-grade gliomas have a poorer prognosis. The average survival period for Grade III is typically between 2-5 years, although a certain proportion of patients can achieve long-term survival (8). GBM is the most malignant form of glioma, and its survival period is usually short, with an average of around 12-15 months (9).

**Figure 1:**
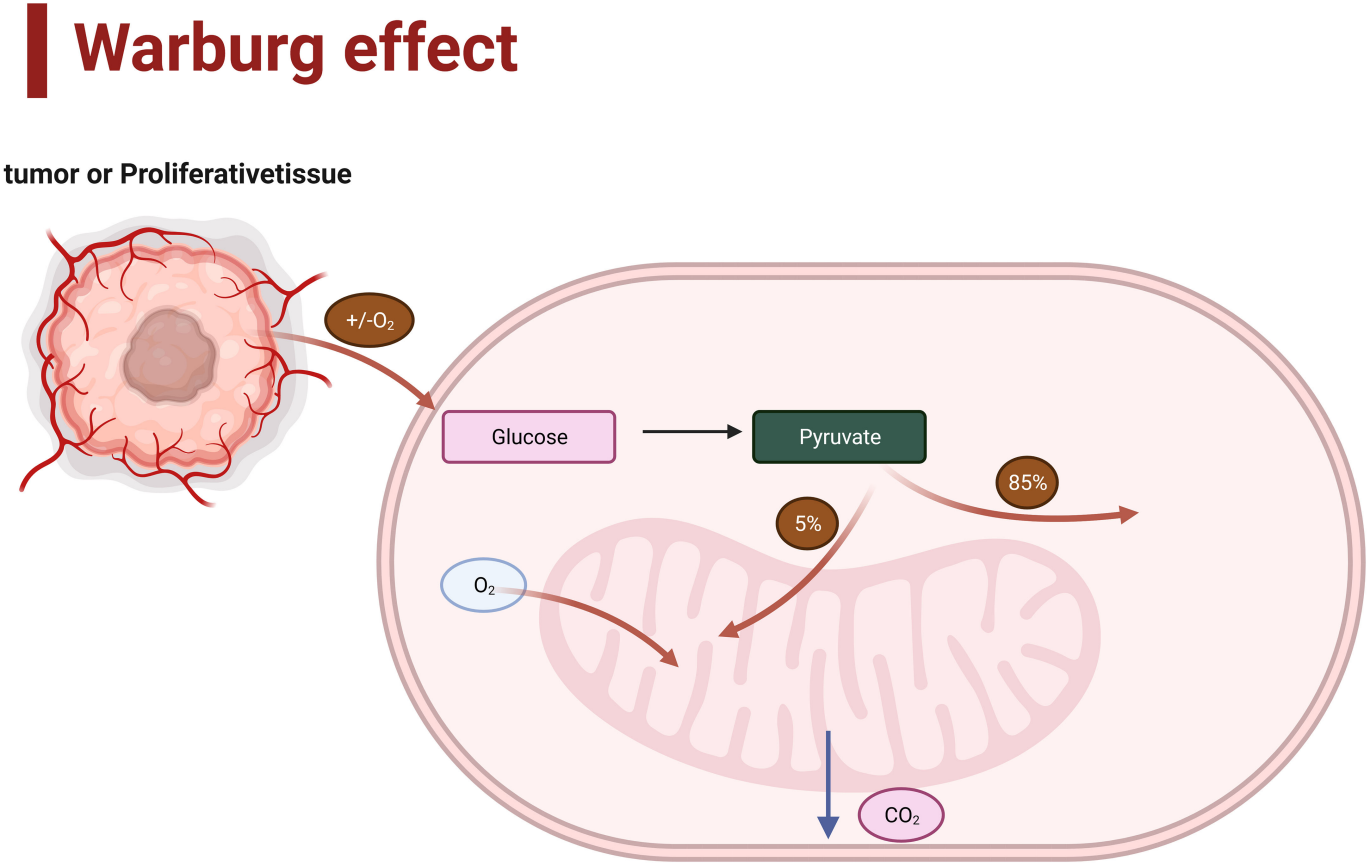
Aerobic glycolysis (Warburg effect).

There is a correlation between the grade of gliomas and their metabolism. Gliomas of different grades may exhibit various features and differences in cellular metabolism (10). IDH (Isocitrate dehydrogenase) is the most crucial molecular diagnostic marker in glioma diagnosis and is used to assist in grading gliomas. For example, IDH1 and IDH2 play essential roles in various cellular functions, including glucose sensing, glutamine metabolism, lipid synthesis, and regulation of cellular redox state (11). Compared to IDH wild-type gliomas, IDH1-mutant gliomas exhibit distinct metabolic and microenvironmental characteristics (12). Moreover, compelling evidence suggests that a key hallmark of malignant progression in gliomas is metabolic reprogramming towards aerobic glycolysis, known as the Warburg effect (13)(**Figure 1**). In addition, the gliomas may show different activation or inhibition of metabolic pathways and accumulation or consumption of metabolites, and malignant gliomas usually accelerate the synthesis of metabolic (14). Therefore, understanding this correlation helps comprehend the biological characteristics of gliomas and provides guidance for developing therapeutic strategies that target specific metabolic pathways.

This comprehensive review systematically summarizes and describes the changes in cellular metabolism across different grades of glioma using a multi-omics analysis approach. Comprehensive analysis at multiple levels, including transcriptomics, proteomics, and metabolomics, can reveal aberrant changes in metabolic pathways, differences in the expression of key regulatory factors, and metabolic features associated with the glioma progression and degree of malignancy in gliomas of different grades. Existing multi-omics analysis strategies can be integrated into glioma metabolic studies to develop a comprehensive and accurate understanding. Moreover, the variability of metabolic characteristics among glioma subtypes can further understand the metabolic remodeling in tumor progression. Therefore, understanding the metabolic profile of different grades of glioma typing can provide important guidance for diagnosing, assessing prognostic, and selecting therapeutic regimens.

### 1.1 Application of multi-omics analysis techniques in glioma metabolism research

The molecular basis of many of these metabolic adaptations is poorly understood. With integration a large amount of multi-omics sequencing data from glioma patients by the Cancer Genome Atlas (TCGA) and the Chinese Glioma Genome Atlas (CGGA), new phenotypic and genomic features were integrated (15, 16). In addition, many publicly available glioma patients were re-analyzed for transcriptional sequencing, protein sequencing, metabolite sequencing, and single-cell and other multi-omics data. Single-cell RNA sequencing has revealed extensive transcriptional cell state diversity in cancer, often observed independently of genetic heterogeneity. Moreover, this raises central questions about how malignant cell states are encoded epigenetically. Studies have been conducted to address this issue using multi-omics single-cell analyses (17). Thus, the study directly compared the epigenetic profiles of different cellular states, revealing key switches for state transitions, recapitulating neurodevelopmental trajectories, and highlighting the aberrant epigenetic mechanisms behind gliomagenesis.

Transcriptomics can reveal key genes and metabolic pathways involved in metabolic regulation in different glioma phenotypes by measuring gene expression levels. For example, metabolomic and transcriptomic analyses of human and mouse GBM showed that the expression of phosphoglycerate dehydrogenase (PHGDH) and serine metabolism were significantly altered in tumor endothelial cells. Moreover, PHGDH is a crucial enzyme in the serine synthesis pathway, and expression of this enzyme serve as a marker for tumor aggressiveness (18). It was also demonstrated that PHGDH-mediated endothelial cell (EC) metabolism promotes hypoxia and immunosuppression in the tumor microenvironment, leading to glioblastoma (GBM) resistance to chimeric antigen receptor T-cell (CAR-T) cellular immunotherapy (19).

Combined metabolomics and proteomics data identified specific lipid distributions in different subtypes and distinct overall metabolic changes in IDH mutant tumors. Designating the three clusters observed in IDH-WT tumors as NMF1 (preganglionic-like; n = 29), NMF2 (mesenchymal-like; n = 37), NMF3 (classical-like; n = 26), prognostic differences were found for type 3 (20). Moreover, NMF1 is enriched in synaptic vesicle recycling and neurotransmitter transport. NMF2 is enriched in innate immune responses, including neutrophil degranulation, phagocytosis, and extracellular matrix organization. NMF3 is enriched in mRNA splicing and RNA metabolism. Therefore, three proteins associated with low tumor survival were identified: low expression of HIST3H2BB, high expression of MT-CYB, and high expression of PRODH (20).

Metabolomics techniques can discern metabolic pathway dysregulation in different grades of gliomas. Given that metabolic reprogramming is a hallmark of cancer, metabolomic analysis can also aid in assessing the progression and malignancy of gliomas. This enables comprehensive monitoring of endogenous metabolites and pathological responses, including genetic alterations (21). By comparing the metabolomic profiles of glioma tumor tissue and adjacent non-tumor tissue, a total of 198 distinct metabolites were identified (22). In addition, these differentially expressed metabolites serve as a foundation for discovering essential metabolic markers and pathways that may be associated with the progression of gliomas (22). Four phosphatidylserines, short-chain acylcarnitine, adenosine, N-methyl-L-glutamate, and sphingolipids were identified among the elevated metabolites. Furthermore, significant metabolic alterations were observed among different gliomas grades, with 17 major metabolites being identified. Among these 17 important metabolites, four acylcarnitines and N-methyl-glutamate showed substantial elevation in grade III/IV compared to grade II glioma tissues. Moreover, these metabolites were significantly higher in glioma tissues than adjacent non-tumor tissues. Additionally, the remaining 12 important metabolites exhibited notable reductions, primarily including lipid species such as lysophosphatidylethanolamines (LPEs), phosphatidylserines (PSs), and phosphatidylinositols (PIs). In summary, the metabolic changes observed between different grades of glioma tissues reveal significant metabolic pathways that are closely associated with the progression of malignant tumor.

### 1.2 Relationship between metabolic changes and glioma classification

Glioma metabolism also depends on the interaction between glioma genotypes and cells in the tumor microenvironment. Moreover, glioma is a complex tumor with multiple gene mutations and diverse metabolic characteristics, resulting in a highly invasive phenotype and poor prognosis. In addition, identifying the key metabolic characteristics that can distinguish glioma subtypes can potentially improve subtype diagnosis and targeted therapy (23). The metabolic phenotype of astrocytomas IDH-Wild type is readily distinguished from glioblastomas IDH-Wild type and oligodendrogliomas mainly because of the low levels of widespread acylcarnitines in the astrocytomas (**Supplementary Table 1**). Moreover, there were significant differences in survival time among the six glioma subtypes of each the correlation and importance of characterizing the specific metabolic characteristics (23). In some studies, 1233 metabolism-related genes were used to cluster the diffuse low-grade glioma cohort in the TCGA database, and three subtypes with different metabolic characteristics were obtained (**Supplementary Table 2**) (24). M1 is predominantly enriched in neurotransmitter secretion, glutamate secretion, and nervous system development.M2 is enriched in immune response, T-cell co-stimulation, cell-cell adhesion, and DNA replication. M3 is significantly enriched in inflammatory response and translation. Finally, the metabolic characteristics with prognostic significance have been identified, and a new way to guide the clinical prediction of the prognosis of diffuse low-grade glioma (25).

The obvious metabolic feature is that IDH wild type and IDH mutant astrocytoma have lower levels of broad-spectrum acylcarnitine than glioblastoma and oligodendroglioma, which indicates that glioblastoma and oligodendroglioma rely more on fatty acid oxidation as an energy source (23). In addition, fatty acid metabolism plays an important role in cell metabolism. Fatty acids can participate in the synthesis of phospholipids and the transduction of necessary signals (such as PI3K/Akt/mTOR) on the membrane of cancer cells (26, 27). Moreover, several studies have discovered that glioma cell subsets after fatty acid-related genotyping are associated with a variety of clinical molecular characteristics (such as age, grade, IDH1 mutation status, 1p19q status, and MGMT status). At the same time, risk scores are good predictors of low-risk patients (28). Two subtypes of human oligodendrogliomas were defined as carbohydrates and fats and associated with comparisons that revealed metabolic differences related to amino acids, carbohydrates, fats, and vitamins. The Oligo2 glioma subtype patients have good prognostic outcomes, possibly due to enhanced immune response and metabolic activation (29). Metabonomics studies the metabolic status by analyzing small molecular metabolites in samples. All showed a more consistent spatial distribution within the tumor, especially the three high acylcarnitines, myristoylcarnitine (m/z 410.2666), palmitoylcarnitine (m/z 400.3422), and stearoylcarnitine (m/z 428.3734), which were significantly stronger at the edge of the tumor than in the tumor interior (30). Therefore, metabonomic analysis of glioma samples can reveal the metabolic differences between different types and identify the abnormal accumulation or depletion of specific metabolites. IDH1 mutations are frequently detected found in low-grade gliomas and secondary glioblastomas. Moreover, a comprehensive metabolomics analysis revealed metabolic reprogramming in human gliomas with IDH1 mutations. Changes in metabolic substances in IDH mutant and IDH wild-type patients were summarized by metabolomic analysis (**Supplementary Table 3**) (23). Compared to IDH1 wild-type gliomas, IDH1 mutant gliomas exhibit significant metabolic changes, shifting from glycolysis to lipid metabolism (31). Finally, we performed WHO2,3,4 grade metabolic signaling pathway enrichment analysis using 693 glioma patients from the Chinese glioma database. Most of the metabolism-related signaling pathways were found to be enriched in WHO2 and WHO4-grade glioma patients (**Supplementary Figure 1**).

### 1.3 Molecular mechanism analysis gliomas changes in gliomas

The constituent cells of the brain, including astrocytes, neurons, and microglia, also interact with each other. This indicates that the interaction between different types of cells is important for the metabolic balance of the brain (32). In addition, glioma cells increase the storage of lipids, amino acids, and nucleotides through various molecular mechanisms, including different pathways ab initio synthesis, and the flow of carbon and nitrogen through multiple pathways (33, 34). Moreover, astrocytes can absorb neurogenic glutamate, and gliomas are produced in this complex and frequently hypoxic metabolic environment. This environment affects the metabolic selection of glioma cells to promote tumor invasive growth (35, 36). Some studies have proved the metabolic changes in glioma and its microenvironment (14, 22, 31, 37–39). Furthermore, an analysis of interstitial fluid from high-grade gliomas and adjacent non-tumor brain tissues also found that the concentration of amino acids in high-grade gliomas was 2 to 8 times higher (40). In addition, the consumption and synthesis of Glycine are associated with rapid cell proliferation in various cancer cells (41). The high glycine synthesis in glioma cells is related to the activation of serine hydroxymethyltransferase (42). GBM has also been shown to utilize metabolites such as fatty acids, glutamine, folic acid, methionine (and their methylated derivatives), urea cycle metabolites, and excessive autophagy processes to meet its high ATP requirements (43). Since the uptake of these metabolites occurs disproportionately in malignant cells, targeting key regulatory molecules in their respective pathways represents a promising approach for cancer treatment. New therapeutic targets to alter tumor metabolism are constantly being discovered, and research in this field should continue.

**Figure 2:**
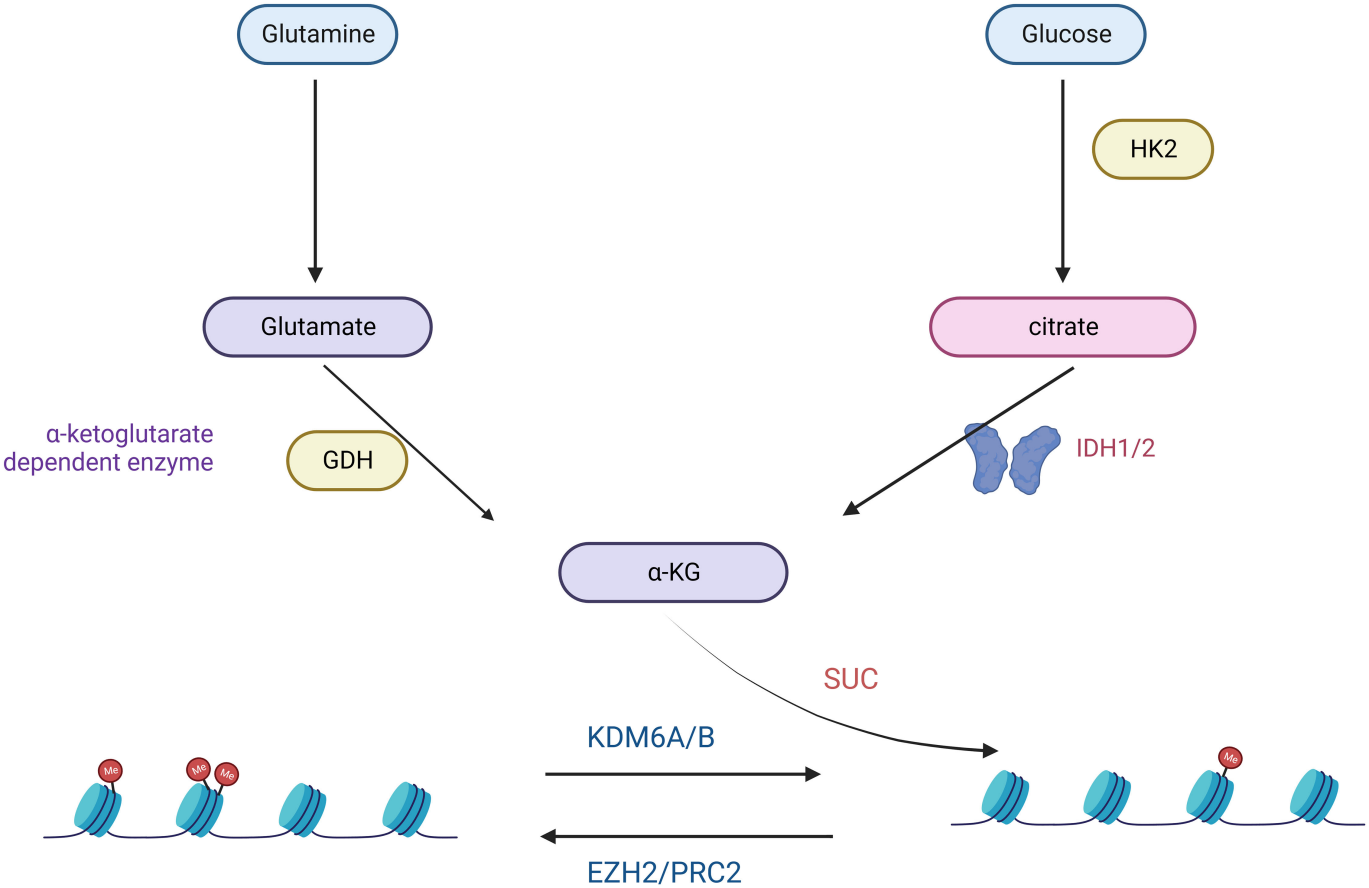
Glutamine- and Glucose-generated α-KG is required for the maintenance of low H3K27me3 levels in H3K27M mutant cells.

A hallmark of many malignancies, including GBM, is an extremely high glutamine (Gln) depletion (44). Glutamine promotes ATP synthesis, redox homeostasis, and biomolecules such as proteins, lipids, and nucleic acids (44). Glutamine and Glucose Starvation of Gliomas Carrying the H3K27M Mutation Significantly Elevates H3K27me3 Levels and Will Dramatically Inhibit Cell Proliferation (45) (**Figure 2**). Several studies have shown that the release of excitotoxic concentrations of glutamate through cystine-glutamate anti-transporter proteins promotes malignant GBM growth (46–48). In malignant cells, the excessive uptake and breakdown metabolism of glutamine serves three primary purposes: facilitating supporting NADPH through anaplerosis in the TCA cycle, supporting a significant increase in glutamate production, facilitating the uptake of essential amino acids (EAAs) through the LAT1 antiporter protein (49). Due to the heavy reliance of GBMs on glutamine uptake, alterations in glutamine metabolism or glutamate signaling have become attractive therapeutic targets. Treatment of C801 glioma cells with the NMDA antagonist MK6 significantly reduces in tumor growth, primarily attributed to decreased glutamate secretion (46). Enzymes involved in fatty acid oxidation, specifically carnitine palmitoyltransferase and long-chain acyl-CoA dehydrogenase, are upregulated in human glioma tissues (50).

In the realm of targeted metabolic therapies, the prevalence of the Warburg effect has drawn attention to targeting glucose metabolism to halt tumor progression (51). Cancer cells support their abnormal proliferation and survival by reprogramming nutrient uptake and metabolism (33). The two main nutrients cancer cells use are the sugar glucose and the amino acid glutamine (52). These two nutrients are central to many anabolic processes, including ATP, nucleotides, proteins, and lipids (53). The proto-oncogene MYC has also emerged as a central regulator of metabolic alterations in gliomas (54, 55). MYC directly controls the expression of glucose metabolism genes, predominantly the glucose transporter GLUT1, hexokinase 2 (HK2), myofructokinase (PFKM), and enolase 1 (ENO1), stimulating the Warburg effect (56, 57). H3K27M diffuse intrinsic pontine glioma (DIPG) is a fatal disease that currently has no effective treatment. Studies have demonstrated that a comprehensive analysis of H3.3K27M cells and tumors shows enhanced glycolysis, glutaminolysis, and tricarboxylic acid cycle metabolism, resulting in high α-ketoglutarate (α-KG) production (45). Inhibiting key enzymes in glycolysis or glutaminolysis increases H3K27me3, which alters chromatin accessibility and extends survival in animal models (45).

Moreover, metabolic adaptation to aerobic glycolysis is a common feature of cancer cells. It is difficult for T cells to infiltrate tumors due to abnormal tumor vasculature, which limits the effectiveness of immunotherapy. Metabolomic and transcriptomic analyses of human and mouse GBM showed that the expression of PHGDH and the metabolism of serine were significantly altered in tumor endothelial cells (19). It has also been investigated that cancer cells can resist cytotoxic T-cell killing by utilizing aerobic glycolysis, which is mediated by key enzymes such as glucose transporter 1, which has been shown to enhance targeting Glut1 enhances T-cell killing (58).

### 1.4 Potential clinical applications treatment strategies

Metabolic biomarkers hold potential in the diagnosis and prognostic assessment of gliomas. The identified differential metabolites serve as promising biomarkers that can be applied for the early detection and prognostic evaluation of gliomas. hey are also important targets for drug development. They can be used to predict the prognosis of patients with brain gliomas through various methods, including genomics, molecular analysis, and multimodal imaging. For example, IDH1 is one of the most common molecular assays for gliomas. It is a cytoplasmic NADP+-dependent enzyme involved in cellular metabolism. IDH1 also predicts patient sensitivity to and as prognosis. In gliomas, PTRF/Cavin-1 not only serves as a marker for patient prognosis, but also promotes malignant behavior by affecting tumor cell endocytosis and exocytosis, reprogramming tumor lipid metabolism, and remodeling the extracellular matrix of tumors (59). The mitochondrial transporter protein receptor (TSPO) is commonly used as a diagnostic tool for gliomas. TSPO regulates glioma cell growth and neovascularization by modulating the metabolic balance between aerobic glycolysis and mitochondrial phosphorylation (60). The epidermal growth factor receptor (EGFR) is gliomas’ most commonly overexpressed protein. The EGFR is a transmembrane glycoprotein that, upon stimulation, leads to the activation of PI3K signaling as well as intracellular MAPK pathways, SRC kinase, and STAT transcription factors (61). These metabolic markers can be used as characteristic indicators in blood or tissue samples to guide personalized management of gliomas.

Multi-omics studies allow for the assessment of used to guide the administration of targeted therapies. Metabolic patterns of glioma patients, against which targeted therapies can be administered. For example, the metabolomic analysis of plasma samples from 87 patients with gliomas revealed that uracil, arginine, and pyroglutamic acid were three metabolites significantly elevated in high-grade gliomas (62). Understanding the metabolic changes in various grades of gliomas provides a foundation for developing specific therapeutic strategies. GBM is characterized by abundant blood vessels, abnormal vascularity, and microvascular proliferation. One study isolated endothelial cells from surgical tumor specimens of glioma patients for metabolomic analysis. Taking the metabolism of endothelial cells as an approach offering an enhanced investigation point, we explored whether targeting tumor endothelial metabolism could improve immunotherapy, thus providing a new therapeutic strategy for anti-tumor therapy (19). By intervening in aberrant metabolic pathways, such as regulating elevated metabolites or restoring reduced metabolites, the growth and spread of tumors can be effectively controlled. These are relevant aspects of potential clinical applications and therapeutic strategies, which are expected to improve the diagnosis and therapeutic outcome of gliomas and promote the development of individualized therapy through the utilization of metabolic markers and therapeutic strategies targeting metabolic pathways. Several targets that influence metabolic signaling pathways in gliomas were summarized (**Supplementary Table 4**).

## 2 Challenges and prospects

**Figure 3:**
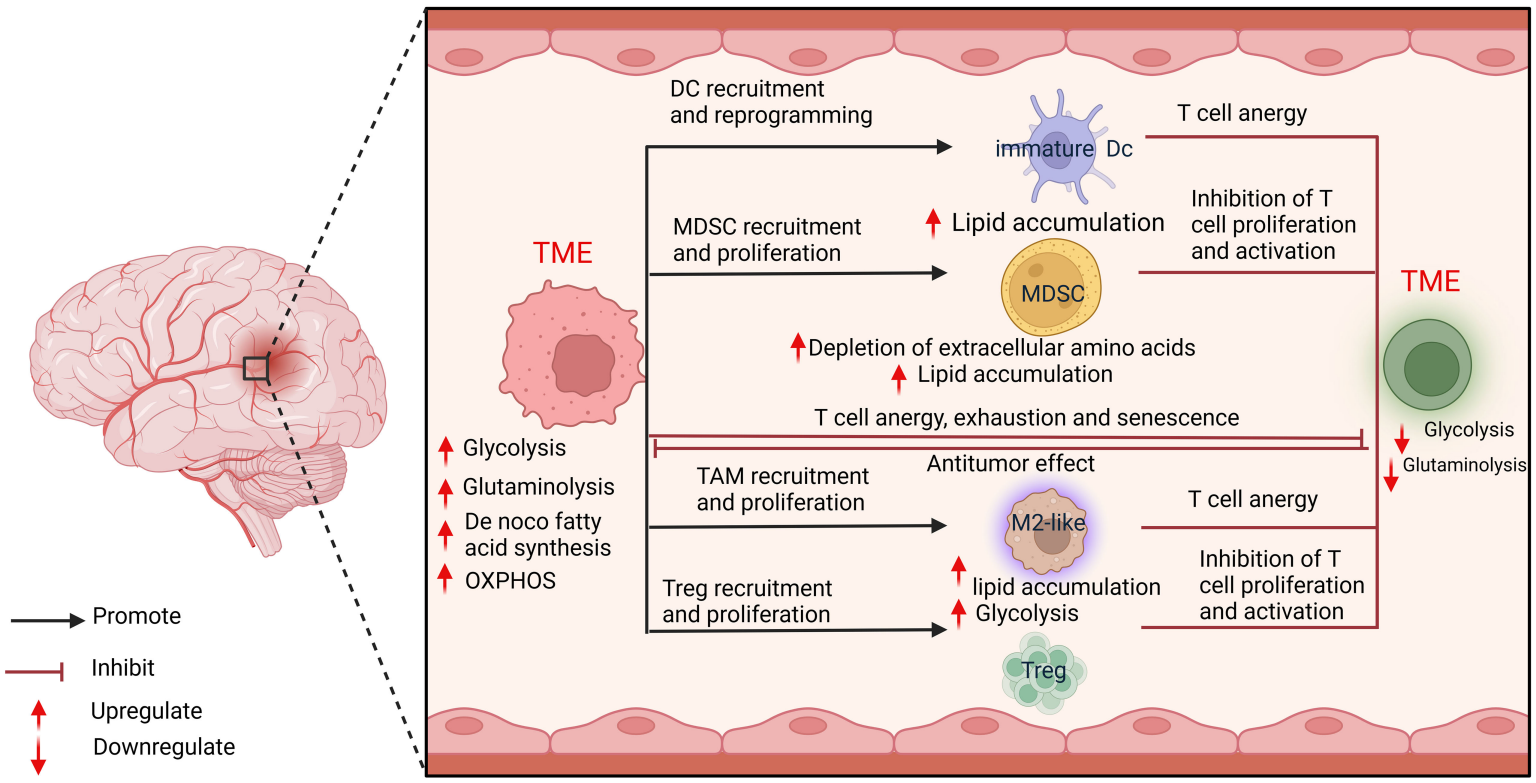
Cellular metabolic interactions between tumor cells and immune cells.

Tumor cells have evolved to use unusual metabolic pathways to enhance their growth as well as to mediate immunosuppression and immune escape. Despite significant advances in understanding gliomas’ molecular pathogenesis and biology, this knowledge has not substantially improved patient prognosis. Accurate detection of the malignant progression of gliomas advancement in their metabolic reprogramming is crucial for developing anti-glioma drugs and glioma therapies and their diagnosis and prognosis. Altered energy metabolism is not unique to cancer cells and can support the bioenergetic needs of various normal cells, including immune cells (63, 64) (**Figure 3**).

Studies have demonstrated that glycosylation, glucose side-branch metabolism, and glycogen metabolism are interconnected in regulating tumor immunosuppression escape. This discovery offers new targets and strategies for next-generation immunotherapy (65–67). Based on metabolomics findings, more personalized treatment strategies can be developed for glioma patients. Predicting efficacy and drug response based on metabolic profiling can enhance treatment success and minimize undesired side effects. Metabolic reprogramming leads to changes in intracellular metabolite levels, which can then influence oncogenic signaling by controlling epigenetics and therefore by globally altering gene transcription (68). Subtypes of adult gliomas can be distinguished by their metabolic characteristics (23). Defining glioma subtypes by metabolic reprogramming opens up new directions for studying therapies with metabolically related mechanisms that do not depend on a single genetic tumor event (25). In addition, many metabolites can be detected by non-invasive imaging techniques, thus enabling the differentiation of glioma subtypes before surgery and providing a basis for non-invasive testing. Through further research, it is expected that more specific and sensitive metabolic markers will be identified to improve the early diagnosis and prognostic assessment of gliomas. This will help provide a more accurate basis for clinical decision-making and guide patients’ treatment options.

To fully understand the relationship between metabolic changes and the development of gliomas, a larger and diversified sample set is needed. This will help to reveal the metabolic differences among different glioma subtypes, grades, and individuals to more accurately identify potential biomarkers and therapeutic targets. Metabolic group sequencing produces a large amount of data, which requires effective data analysis and interpretation methods. The integration of metabonomics data with other genomics data (such as genomics and transcriptome) can provide a more comprehensive map of the glioma metabolic network and further reveal the complex regulation mechanism of metabolic pathways. Glioma is a complex and diversified tumor, which is affected by a variety of genetic and environmental factors in its development. Solving the challenges of glioma metabolism requires the cooperation of interdisciplinary teams, including experts in the fields of biology, medicine, computer science, and statistics, to study and solve related problems.

Based on the results of metabonomics, more individualized treatment strategies can be developed for glioma patients. By predicting efficacy and drug reactions according to metabolic characteristics, the success rate of treatment can be improved and unnecessary side effects can be reduced. New biomarkers: further research, it is expected to find more specific and sensitive metabolic markers to improve the early diagnosis and prognosis. This will help to provide a more accurate basis for clinical decision-making and guide patients’ treatment plans.

## 3 Conclusions

In summary, it offers promising applications and important potential application prospects in glioma research by overcoming gliomas. This will enable us to offer a personalized approaches the abnormal metabolism, gain a deeper understanding of the aim of various challenges and cooperate with experts in different fields; we are expected better to understand the mechanism of abnormal metabolism of gliomas and provide more individual methods and guidance for early diagnosis, treatment, and prognosis evaluation of gliomas.

## Author contributions

XY: Conceptualization, Methodology, Writing – original draft, Writing – review & editing. JL: Conceptualization, Investigation, Methodology, Project administration, Supervision, Writing – original draft, Writing – review & editing. YZ: Conceptualization, Investigation, Methodology, Project administration, Writing – review & editing. CL: Methodology, Writing – review & editing. PL: Methodology, Writing – review & editing. TL: Data curation. Writing – review & editing. QL: Methodology, Writing – review & editing. XH: Conceptualization, Validation, Writing – original draft.
