# Corrigendum: Alterations in cellular metabolism under different grades of glioma staging identified based on a multi-omics analysis strategy

**DOI:** 10.3389/fendo.2024.1486498

**Published:** 2024-09-26

**Authors:** Xianlei Yan, Jinwei Li, Yang Zhang, Cong Liang, Pengcheng Liang, Tao Li, Quan Liu, Xuhui Hui

**Affiliations:** ^1^ Department of Neurosurgery, West China Hospital, Sichuan University, Chengdu, Sichuan, China; ^2^ Department of Neurosurgery, Liuzhou Workers Hospital, Liuzhou, Guangxi, China; ^3^ Department of Vascular Surgery, Fuwai Yunnan Cardiovascular Hospital, Affiliated Cardiovascular Hospital of Kunming Medical University, Kunming, Yunnan, China; ^4^ Department of Pharmacy, Liuzhou Workers Hospital, Liuzhou, Guangxi, China; ^5^ Department of Medical Imaging, Liuzhou Workers Hospital, Liuzhou, Guangxi, China

**Keywords:** multi-omics analysis, cellular metabolism, glioma, single-cell, biomarker

In the published article, there was an error in Supplementary Tables 1 and 3. The copied tables did not contain a reference to the original research paper that they were copied from Björkblom et al., 2022.

The authors apologize for this error and state that this does not change the scientific conclusions of the article in any way. The original article has been updated.

