# Supplementary material for: Alterations in cellular metabolism under different grades of glioma staging identified based on a multi-omics analysis strategy
Source: Front Endocrinol (Lausanne). 2023 Dec 4;14:1292944. doi: 10.3389/fendo.2023.1292944 (PMC10726964; doi:10.3389/fendo.2023.1292944)
Supplement: Supplementary file 1 [file DataSheet1.zip › Supplement Table 1.docx]

| **Supplementary Table 1**. Metabolites with consistently higher or lower levels in; WHO grade 2 and 3 astrocytoma, IDH-mutant; WHO grade 2 and 3 oligodendroglioma, IDH-mutant and 1p19q-codeleted; and WHO grade 4 glioblastoma, IDH-mutant^1^.  **Panel A.** Metabolites with consistently higher (↑) or lower (↓) levels in glioblastoma, IDH-mutant tumors compared to IDH-mutated astrocytoma and oligodendroglioma. P<0.01 was required for all metabolites in each subtype comparison. >2-fold difference was required in at least one subtype comparison. | | | | | |
| --- | --- | --- | --- | --- | --- |
| **Named metabolite** | **Levels in GBM IDH mut** | **P-value,**  **Astro vs. GBM** | **Fold change, Astro / GBM** | **P-value, Oligo vs.**  **GBM** | **Fold change, Oligo / GBM** |
| N-acetyl Aspartic acid (NAA) | ↓ | 5.2E-03 | 5.74 | 3.2E-05 | 7.67 |
| Aldopentose (Xyl/Lyx/Ara/Rib) ^#2^ | ↓ | 3.5E-03 | 2.81 | 1.9E-04 | 2.82 |
|  |  |  |  |  |  |
| Glycine | ↑ | 2.9E-05 | 0.19 | 5.9E-07 | 0.30 |
| 2-Aminoadipic acid | ↑ | 2.5E-04 | 0.26 | 2.8E-05 | 0.36 |
| Asparagine | ↑ | 3.1E-03 | 0.31 | 2.6E-04 | 0.40 |
| Ornithine (Arginine/Citrulline) | ↑ | 2.3E-05 | 0.17 | 1.9E-06 | 0.44 |
| Phenylalanylproline | ↑ | 3.4E-03 | 0.40 | 2.4E-05 | 0.54 |
| Proline | ↑ | 1.3E-03 | 0.25 | 1.0E-04 | 0.54 |
| Threonine | ↑ | 4.9E-04 | 0.36 | 5.8E-03 | 0.60 |
| Tyrosine | ↑ | 3.7E-04 | 0.40 | 1.6E-05 | 0.61 |
| Methionine | ↑ | 1.6E-03 | 0.40 | 4.7E-04 | 0.63 |
| Valine | ↑ | 2.5E-04 | 0.43 | 3.7E-03 | 0.67 |
| Isoleucine/Leucine | ↑ | 1.1E-04 | 0.48 | 2.9E-03 | 0.72 |
| Hypotaurine | ↑ | 2.1E-03 | 0.29 | 8.6E-03 | 0.76 |
| Valerylcarnitine (C5:0) | ↑ | 3.6E-03 | 0.23 | 6.7E-03 | 0.42 |
| Octenoylcarnitine (C8:1) | ↑ | 4.0E-03 | 0.38 | 9.4E-06 | 0.53 |
| Linolenate (18:3) | ↑ | 2.8E-03 | 0.41 | 3.0E-04 | 0.39 |
| Eicosatrienoic acid 5,8,11 (Mead acid, 20:3) | ↑ | 4.0E-03 | 0.43 | 2.4E-03 | 0.55 |
| 2-Hydroxypalmitate | ↑ | 3.8E-03 | 0.42 | 7.5E-03 | 0.60 |
| 2-Hydroxyhexanoate | ↑ | 6.4E-06 | 0.47 | 7.7E-06 | 0.61 |
| 1-pentadecanoylglycerophosphocholine (15:0) | ↑ | 1.8E-04 | 0.50 | 2.3E-03 | 0.72 |
| gamma-Glutamylmethionine | ↑ | 7.1E-04 | 0.34 | 9.4E-05 | 0.55 |
| gamma-Glutamylleucine/isoleucine | ↑ | 4.4E-03 | 0.37 | 4.7E-03 | 0.56 |
| gamma-Glutamyltyrosine | ↑ | 2.0E-03 | 0.41 | 7.1E-04 | 0.60 |
| Disaccharides, Glc-GLC, (Cellobiose/Laminaribiose/Maltose) | ↑ | 1.3E-05 | 0.28 | 3.2E-04 | 0.45 |
| Indolelactate | ↑ | 6.2E-04 | 0.40 | 3.0E-03 | 0.55 |
| 5,6-Dihydrouracil | ↑ | 5.0E-03 | 0.48 | 5.8E-05 | 0.46 |
| Chenodeoxycholic acid glycine conjugate | ↑ | 3.4E-04 | 0.25 | 3.0E-03 | 0.50 |
| Fumaric acid | ↑ | 2.0E-03 | 0.40 | 7.3E-03 | 0.73 |
| **Panel B.** Metabolites with consistently higher (↑) or lower (↓) levels in IDH-mutated astrocytoma compared to IDH-mutated oligodendroglioma and glioblastoma. P<0.01 was required for all metabolites in each subtype comparison. >2-fold difference was required in at least one subtype comparison. | | | | | |
| **Named metabolite** | **Levels in Astro IDH mut** | **P-value,**  **Astro vs. GBM** | **Fold change, Astro / GBM** | **P-value, Astro vs. Oligo** | **Fold change, Astro / Oligo** |
| Eicoseneoylcarnitine (C20:1) | ↓ | 5.6E-04 | 0.12 | 5.4E-07 | 0.12 |
| Hexanoylcarnitine (C6:0) | ↓ | 7.7E-04 | 0.26 | 2.9E-04 | 0.38 |
| Hydroxylauroylcarnitine (C12:0-OH) | ↓ | 6.2E-03 | 0.25 | 7.5E-03 | 0.13 |
| Hydroxylinoleoylcarnitine (C18:2-OH) | ↓ | 8.9E-03 | 0.37 | 1.1E-03 | 0.18 |
| Hydroxyoctadecenoylcarnitine (C18:1-OH) | ↓ | 4.4E-03 | 0.20 | 6.9E-05 | 0.15 |
| Propionylcarnitine (C3:0) | ↓ | 5.3E-03 | 0.18 | 6.8E-03 | 0.53 |
| Stearoylcarnitine (C18:0) | ↓ | 3.6E-03 | 0.16 | 1.1E-04 | 0.23 |
| 1-arachidonoyl-GPC (20:4) | ↓ | 6.1E-03 | 0.46 | 2.9E-06 | 0.65 |
| 1-eicosatrienoyl-GPC (20:3) | ↓ | 3.6E-03 | 0.41 | 5.3E-03 | 0.73 |
| 1-myristoyl-GPC (14:0) | ↓ | 2.1E-06 | 0.35 | 1.2E-06 | 0.52 |
| Glycoursodeoxycholic acid | ↓ | 1.3E-03 | 0.18 | 1.6E-03 | 0.31 |
| 2-Aminobutyric acid (AABA) | ↓ | 3.7E-03 | 0.45 | 2.3E-04 | 0.55 |
| beta-Alanine | ↓ | 8.0E-03 | 0.41 | 9.0E-03 | 0.57 |
| Dimethylarginine (ADMA + SDMA) | ↓ | 1.6E-03 | 0.45 | 1.7E-03 | 0.65 |
| gamma-Glutamyltryptophan | ↓ | 7.7E-03 | 0.48 | 2.7E-03 | 0.59 |
| Lysine | ↓ | 1.3E-04 | 0.33 | 8.3E-05 | 0.42 |
| N-acetylvaline | ↓ | 5.5E-05 | 0.47 | 3.5E-04 | 0.57 |
| Sulfolithocholylglycine | ↓ | 2.9E-03 | 0.42 | 1.1E-03 | 0.63 |
| Glycocholic acid | ↓ | 1.2E-03 | 0.48 | 1.5E-03 | 0.56 |
| O-Phosphoetanolamine | ↓ | 4.0E-03 | 0.38 | 8.7E-05 | 0.46 |
| Uridine 5'-diphospho-N-acetylglucosamine | ↓ | 2.1E-03 | 0.47 | 2.2E-04 | 0.68 |
|  |  |  |  |  |  |
| **Panel C**. In addition to panel B, named metabolites with higher (↑) or lower (↓) levels in astrocytoma, IDH-mutant compared to oligodendroglioma, IDH-mutant. P<0.01 and >2-fold difference was required for all metabolites. | | | | | |
| **Named metabolite** | **Levels in Astro IDH mut.** |  |  | **P-value, Astro vs. Oligo** | **Fold change, Astro / Oligo** |
| Cervonylcarnitine (C22:6) | ↓ |  |  | 3.6E-05 | 0.12 |
| Hydroxypalmitoleoylcarnitine (C16:1-OH) | ↓ |  |  | 2.2E-03 | 0.17 |
| Eicosadieneoycarnitine (C20:2) | ↓ |  |  | 8.7E-05 | 0.19 |
| Linoleoylcarnitine (C18:2) | ↓ |  |  | 1.0E-03 | 0.24 |
| Oleoylcarnitine (C18:1) | ↓ |  |  | 2.1E-04 | 0.24 |
| Linoleneoylcarnitine (C18:3) | ↓ |  |  | 1.2E-03 | 0.25 |
| Myristoylcarnitine (C14:0) | ↓ |  |  | 2.5E-04 | 0.31 |
| Palmitoylcarnitine (C16:0) | ↓ |  |  | 7.4E-04 | 0.33 |
| Hexadecenoylcarnitine (C16:1) | ↓ |  |  | 1.4E-03 | 0.35 |
| Dodecenoylcarnitine (C12:1) | ↓ |  |  | 1.2E-03 | 0.37 |
| Laurylcarnitine (C12:0) | ↓ |  |  | 1.7E-03 | 0.40 |
| Hexadecadienoylcarnitine (C16:2) | ↓ |  |  | 3.8E-03 | 0.44 |
| Tetradecadienylcarnitine (C14:2) | ↓ |  |  | 6.2E-03 | 0.48 |
| Myristoleoylcarnitine (C14:1) | ↓ |  |  | 6.9E-03 | 0.49 |
| Note, no metabolite were consistently changed when comparing IDH-mutated oligodendroglioma vs. astrocytoma and glioblastoma, using P<0.01 for all metabolites and at least 2-fold difference in at least one comparison. | | | | | |

**Reference**

1. Björkblom B, Wibom C, Eriksson M, et al. Distinct metabolic hallmarks of WHO classified adult glioma subtypes. Neuro-oncology 2022;24:1454-1468.
