# Supplementary material for: Alterations in cellular metabolism under different grades of glioma staging identified based on a multi-omics analysis strategy
Source: Front Endocrinol (Lausanne). 2023 Dec 4;14:1292944. doi: 10.3389/fendo.2023.1292944 (PMC10726964; doi:10.3389/fendo.2023.1292944)
Supplement: Supplementary file 1 [file DataSheet1.zip › Supplement table 3.docx]

| **Supplementary Table 3.** Metabolites with higher or lower levels in IDH-mutated tumors compared to IDH-wildtype tumors, irrespective of glioma subtype, i.e. combining astrocytoma, oligodendroglioma and glioblastoma. Named metabolites with higher (↑) or lower (↓) levels in IDH-mutated compared to IDH-wildtype tumors are listed. P<0.01 and >2-fold difference was required for all metabolites^1^. | | | |
| --- | --- | --- | --- |
| **Named metabolite** | **Levels in**  **IDH mut** | **P-value,**  **IDH mut vs. IDH WT** | **Fold change, IDH mut /**  **IDH WT** |
| 2-Hydroxyglutaric acid | ↑ | 2.5E-104 | 157.8 |
| Xylitol/Arabitol/Ribitol | ↑ | 8.7E-33 | 3.73 |
| Erythritol | ↑ | 1.4E-45 | 3.32 |
| Aldopentose (Xyl/Lyx/Ara/Rib) ^#1^ | ↑ | 8.8E-44 | 3.21 |
| Ketohexoses (Fructose) | ↑ | 6.0E-22 | 2.59 |
| Methylmalonylcarnitine /Succinylcarnitine (C4:0) | ↑ | 5.7E-25 | 2.33 |
| myo-Inositol | ↑ | 1.8E-32 | 2.23 |
| Glycerol-3-Phosphate | ↑ | 2.9E-28 | 2.16 |
| Glycerol-2-Phosphate | ↑ | 7.0E-28 | 2.07 |
| Glyceric acid | ↑ | 1.4E-22 | 2.05 |
| Hexadecenoyl-carnitine (C16:1) | ↑ | 4.4E-09 | 2.04 |
|  |  |  |  |
| Sphingosine | ↓ | 3.2E-22 | 0.26 |
| beta-Sitosterol | ↓ | 3.6E-16 | 0.27 |
| 5,6-Dihydrouracil | ↓ | 8.5E-26 | 0.29 |
| 1-stearoylplasmenylethanolamine | ↓ | 2.7E-32 | 0.30 |
| Chenodeoxycholic acid glycine conjugate | ↓ | 1.5E-29 | 0.32 |
| Deoxycholic acid | ↓ | 1.4E-14 | 0.32 |
| 2-Aminoadipic acid | ↓ | 7.9E-11 | 0.33 |
| Phenylacetylglutamine | ↓ | 5.1E-06 | 0.33 |
| Glycoursodeoxycholic acid | ↓ | 4.5E-16 | 0.34 |
| 2-stearoyl-GPC (18:0)/1-stearoyl-GPC (18:0) | ↓ | 9.4E-28 | 0.34 |
| 3-Carboxy-4-methyl-5-propyl-2-furanpropanoate (CMPF) | ↓ | 2.2E-13 | 0.35 |
| Eicoseneoylcarnitine (C20:1) | ↓ | 9.1E-27 | 0.36 |
| Glycocholic acid | ↓ | 2.3E-10 | 0.37 |
| 1-arachidoyl-GPC (20:0) | ↓ | 1.8E-20 | 0.38 |
| Campesterol | ↓ | 1.7E-11 | 0.38 |
| Propionylcarnitine (C3:0) | ↓ | 8.2E-26 | 0.38 |
| Stearoylcarnitine (C18) | ↓ | 6.1E-34 | 0.38 |
| 1-heptadecanoyl-GPC (17:0) | ↓ | 4.7E-34 | 0.42 |
| Cholic acid | ↓ | 4.5E-09 | 0.44 |
| Isoleucylisoleucine | ↓ | 2.6E-08 | 0.44 |
| Uracil | ↓ | 2.9E-18 | 0.46 |
| Sulfolithocholylglycine | ↓ | 7.5E-17 | 0.47 |
| 3-Hydroxydecanoate | ↓ | 5.1E-23 | 0.47 |
| 2-Hydroxypalmitate | ↓ | 6.6E-12 | 0.47 |
| 1-docosahexaenoyl-GPC (22:6) | ↓ | 7.2E-25 | 0.47 |
| Alanine | ↓ | 4.0E-27 | 0.48 |
| 3-Hydroxylaurate | ↓ | 9.5E-24 | 0.48 |
| 1-nonadecanoylglycerophosphocholine(19:0) | ↓ | 3.2E-18 | 0.48 |
| Ornithine (Arginine/Citrulline) | ↓ | 3.4E-35 | 0.49 |
| Asparagine | ↓ | 2.3E-33 | 0.50 |

**References**

1. Björkblom B, Wibom C, Eriksson M, et al. Distinct metabolic hallmarks of WHO classified adult glioma subtypes. Neuro-oncology 2022;24:1454-1468.

The authors apologize for this error and state that this does not change the scientific conclusions of the article in any way. The original article has been updated.
