# Supplementary material for: Alterations in cellular metabolism under different grades of glioma staging identified based on a multi-omics analysis strategy
Source: Front Endocrinol (Lausanne). 2023 Dec 4;14:1292944. doi: 10.3389/fendo.2023.1292944 (PMC10726964; doi:10.3389/fendo.2023.1292944)
Supplement: Supplementary file 1 [file DataSheet1.zip › Supplement Table 4.docx]

| Effects of Silencing | Target | Cellular Process |
| --- | --- | --- |
| Reverses Warburg effect, promoting apoptosis and reducing tumor invasiveness[1]. | PDK1 | Glycolysis |
| Etomoxir inhibits fatty acid oxidation and promotes intracellular ROS accumulation[2]. | CPT-1 | Fatty Acid Oxidation |
| Reduces proliferation of ASS1 silenced cells | CAD | Urea Cycle |
| NMDA receptor antagonist MK508 reduces tumor growth[3]. | GRIN | Glutamate-Glutamine |
| Inhibits autophagosome formation[4]. | ATG7 | Autophagy |

[1] K.K. Velpula, A. Bhasin, S. Asuthkar, and A.J. Tsung, Combined targeting of PDK1 and EGFR triggers regression of glioblastoma by reversing the Warburg effect. Cancer Res 73 (2013) 7277-89.

[2] L.S. Pike, A.L. Smift, N.J. Croteau, D.A. Ferrick, and M. Wu, Inhibition of fatty acid oxidation by etomoxir impairs NADPH production and increases reactive oxygen species resulting in ATP depletion and cell death in human glioblastoma cells. Biochim Biophys Acta 1807 (2011) 726-34.

[3] T. Takano, J.H. Lin, G. Arcuino, Q. Gao, J. Yang, and M. Nedergaard, Glutamate release promotes growth of malignant gliomas. Nat Med 7 (2001) 1010-5.

[4] Y.L. Hu, M. DeLay, A. Jahangiri, A.M. Molinaro, S.D. Rose, W.S. Carbonell, and M.K. Aghi, Hypoxia-induced autophagy promotes tumor cell survival and adaptation to antiangiogenic treatment in glioblastoma. Cancer Res 72 (2012) 1773-83.

Supplement Table 4. Promising therapeutic targets in tumor metabolism.
