# Supplementary figures and images for: Alterations in cellular metabolism under different grades of glioma staging identified based on a multi-omics analysis strategy
Source: Front Endocrinol (Lausanne). 2023 Dec 4;14:1292944. doi: 10.3389/fendo.2023.1292944 (PMC10726964; doi:10.3389/fendo.2023.1292944)

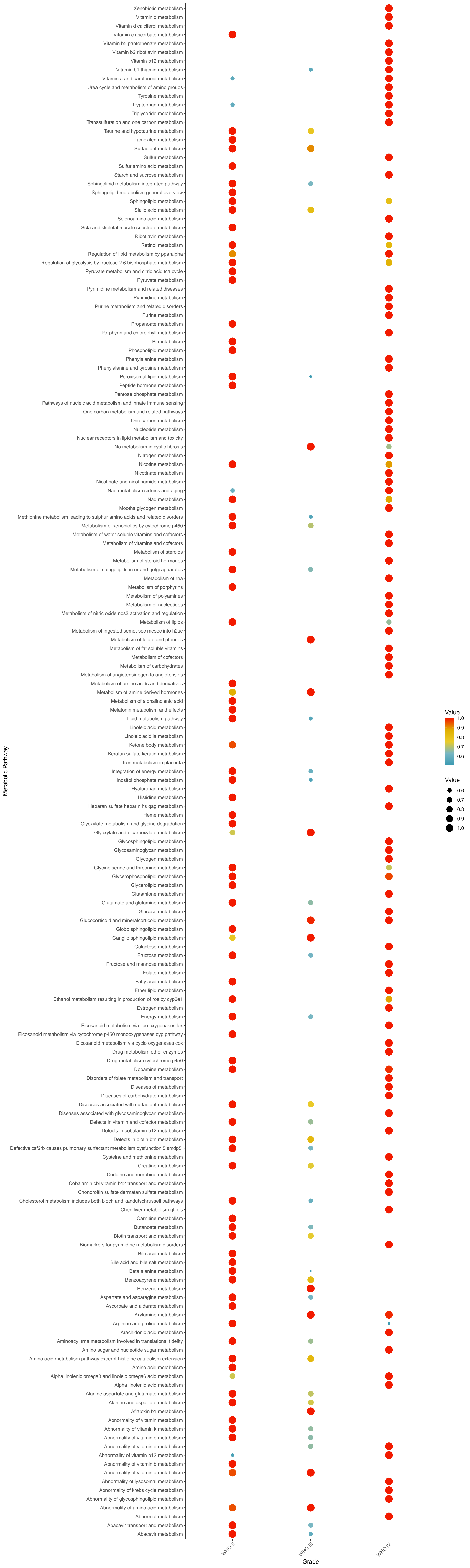

Supplement: Supplementary file 1 [file DataSheet1.zip › Supplement Figure 1.pdf]
